# A Novel Repressor of the *ica* Locus Discovered in Clinically Isolated Super-Biofilm-Elaborating *Staphylococcus aureus*

**DOI:** 10.1128/mBio.02282-16

**Published:** 2017-01-31

**Authors:** Liansheng Yu, Junzo Hisatsune, Ikue Hayashi, Nobuyuki Tatsukawa, Yusuke Sato’o, Emiri Mizumachi, Fuminori Kato, Hideki Hirakawa, Gerald B. Pier, Motoyuki Sugai

**Affiliations:** a Department of Bacteriology, Hiroshima University Graduate School of Biomedical and Health Sciences, Hiroshima City, Hiroshima, Japan; b Project Research Center for Nosocomial Infectious Diseases, Hiroshima University, Hiroshima City, Hiroshima, Japan; c Research Facility, Hiroshima University Faculty of Dentistry, Hiroshima City, Hiroshima, Japan; d Genome Informatics Group, Department of Technology Development, Kazusa DNA Research Institute, Kisarazu City, Chiba, Japan; e Division of Infectious Diseases, Department of Medicine, Brigham and Women’s Hospital and Harvard Medical School, Boston, Massachusetts, USA; MedImmune

## Abstract

*Staphylococcus aureus* TF2758 is a clinical isolate from an atheroma and a super-biofilm-elaborating/polysaccharide intercellular adhesin (PIA)/poly-*N*-acetylglucosamine (PNAG)-overproducing strain (L. Shrestha et al., Microbiol Immunol 60:148–159, 2016, https://doi.org/10.1111/1348-0421.12359). A microarray analysis and DNA genome sequencing were performed to identify the mechanism underlying biofilm overproduction by TF2758. We found high transcriptional expression levels of a 7-gene cluster (*satf2580* to *satf2586*) and the *ica* operon in TF2758. Within the 7-gene cluster, a putative transcriptional regulator gene designated *rob* had a nonsense mutation that caused the truncation of the protein. The complementation of TF2758 with *rob* from FK300, an *rsbU*-repaired derivative of *S. aureus* strain NCTC8325-4, significantly decreased biofilm elaboration, suggesting a role for *rob* in this process. The deletion of *rob* in non-biofilm-producing FK300 significantly increased biofilm elaboration and PIA/PNAG production. In the search for a gene(s) in the 7-gene cluster for biofilm elaboration controlled by *rob*, we identified open reading frame (ORF) SAOUHSC_2898 (*satf2584*). Our results suggest that ORF SAOUHSC_2898 (*satf2584*) and *icaADBC* are required for enhanced biofilm elaboration and PIA/PNAG production in the *rob* deletion mutant. Rob bound to a palindromic sequence within its own promoter region. Furthermore, Rob recognized the TATTT motif within the *icaR-icaA* intergenic region and bound to a 25-bp DNA stretch containing this motif, which is a critically important short sequence regulating biofilm elaboration in *S. aureus*. Our results strongly suggest that Rob is a long-sought repressor that recognizes and binds to the TATTT motif and is an important regulator of biofilm elaboration through its control of SAOUHSC_2898 (SATF2584) and Ica protein expression in *S. aureus*.

## IMPORTANCE

During the search for molecular mechanisms underlying biofilm overproduction of *Staphylococcus aureus* TF2758, we found a putative transcriptional regulator gene designated *rob* within a 7-gene cluster showing a high transcriptional expression level by microarray analysis. The deletion of *rob* in non-biofilm-producing FK300, an *rsbU*-repaired derivative of NCTC8325-4, significantly increased biofilm elaboration and PIA/PNAG production. The search for a gene(s) in the 7-gene cluster for biofilm elaboration controlled by *rob* identified ORF SAOUHSC_2898. Besides binding to its own promoter region to control ORF SAOUHSC_2898 expression, Rob recognized the TATTT motif within the *icaR-icaA* intergenic region and bound to a 25-bp DNA stretch containing this motif, which is a critically important short sequence regulating biofilm elaboration in *S. aureus*. Our results strongly suggest that Rob is a long-sought repressor that recognizes and binds to the TATTT motif and is a new important regulator of biofilm elaboration through its control of SAOUHSC_2898 and Ica protein expression in *S. aureus*.

## INTRODUCTION

*Staphylococcus aureus* is among the most common human pathogens, causing a wide range of infections, from superficial skin and mucosal infections to bone or lung infections, as well as serious systemic diseases. *S. aureus* colonization has been regarded as a risk factor for developing subsequent infections. Some chronic infections, such as endocarditis, osteomyelitis, and those on implanted medical devices, are characteristically associated with biofilm elaboration (1–3). Development of biofilms has been divided into at least three physiologically different stages: initial attachment, biofilm maturation, and detachment (or dispersal), which involves specific factors (4). The matrix of a staphylococcal biofilm is mainly composed of polysaccharides, cell surface and secreted bacterial proteins, and extracellular DNA (5). Cells encased in the matrix are protected from antibiotic therapy and host immune responses (3, 4, 6). Dispersal of cells from a biofilm may be important for the dissemination of the bacteria (7).

The main exopolysaccharide of the *S. aureus* biofilm matrix is poly-*N*-acetylglucosamine (PNAG), which is also known as polysaccharide intercellular adhesin (PIA) (8). The synthesis and accumulation of PIA/PNAG on the cell surface are carried out by the products of four genes: *icaA*, *icaD*, *icaB*, and *icaC* (9). These genes are located in one operon and were first identified by Heilmann et al. (10). Recent studies have indicated that the expression of *icaADBC* is affected by a number of regulatory and environmental factors (11–14). The *icaR* gene is located adjacent to *icaADBC* but is divergently transcribed from this operon (15). The protein encoded by *icaR* belongs to the TetR family of transcriptional regulators and represses *icaADBC* transcription by binding to a region immediately upstream of the *icaA* start codon (16). Additionally, environmental factors, including glucose, ethanol, high temperatures, and high osmolarity, have been reported to affect biofilm elaboration (11–14). Ethanol increases the expression of *icaA* by repressing *icaR* transcription (15). In contrast, enhancement of *icaA* expression by high glucose or NaCl levels was found to occur independently of *icaR*.

A 5-nucleotide motif (TATTT) within the *icaR-icaA* intergenic region was previously shown to play a key role in the transcription of the *ica* locus (16). This study also demonstrated that IcaR binds to a 42-bp sequence within the *ica* promoter region but not the TATTT sequence. Hence, the effects of the TATTT motif on *icaADBC* expression have been suggested to be controlled by another, as-yet-unidentified repressor(s).

We evaluated the biofilm-elaborating ability of clinical isolates in Japan and found that TF2758, which was isolated from an atheroma, is an extremely high biofilm producer (17). Whole-genome sequencing and a microarray analysis of TF2758 discovered a spontaneous mutation in a putative transcriptional regulator gene, within a 7-gene cluster, which was expressed at markedly higher levels than in a non-biofilm-elaborating control strain. We designated this gene *rob*, *r*egulator *o*f *b*iofilm. In the present study, we demonstrate that Rob is a long-sought repressor that recognizes and binds to the TATTT motif and suggest that Rob is an important regulator of biofilm elaboration through its control of the expression of as-yet-uncharacterized hypothetical protein SAOUHSC_2898 (SATF2584) and IcaADBC.

## RESULTS

### Identification of *rob* from a super-biofilm-elaborating strain.

**FIG 1:**
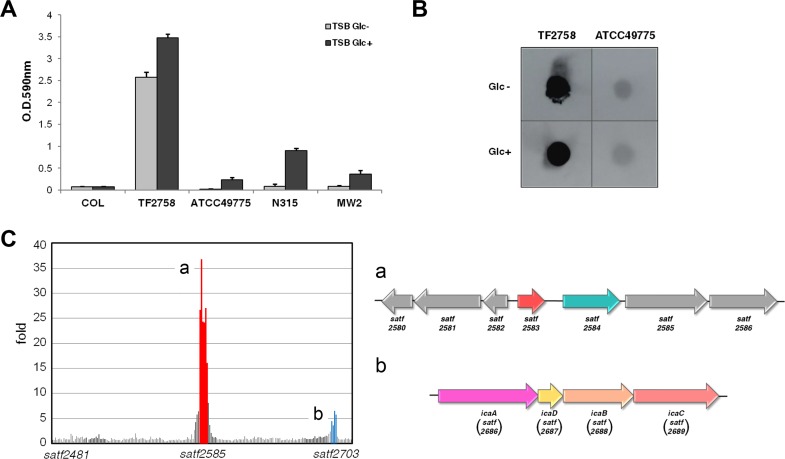
Biofilm elaboration and PIA/PNAG production by *Staphylococcus aureus* TF2758. (A) Biofilm elaboration. Bacteria were grown in trypticase soy broth (TSB) in the presence (Glc+) or absence (Glc-) of 1% glucose. Biofilm elaboration was measured using the polystyrene microtiter plate assay described in Materials and Methods. The averages and standard errors for each sample are shown. (B) PIA/PNAG production. Extracts from overnight cultures were spotted on a membrane, and PIA/PNAG was detected by rabbit anti-PNAG, as described in Materials and Methods. Non-biofilm-elaborating strain ATCC 49775 was used as a control in the comparative microarray analysis. Figures were compiled from separate images on the same film. (C) Comparative gene expression analysis of TF2758 and ATCC 49775. TF2758 gene expression was represented as a fold change from that of ATCC 49775. Two gene clusters exhibiting marked increases in gene expression are colored (red and blue), and these gene clusters are depicted at right (a and b).

**FIG 2:**
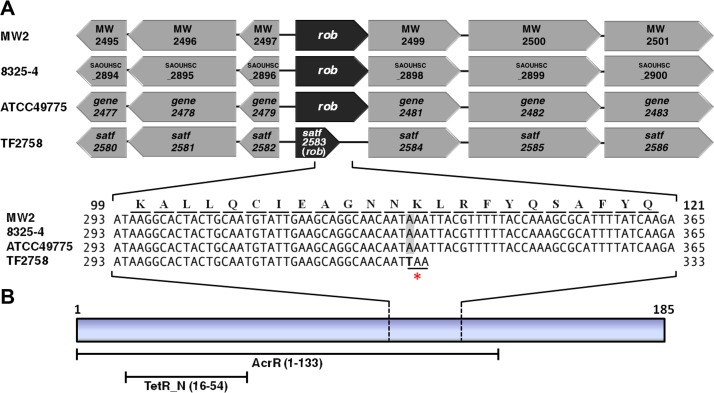
Identification of a nonsense mutation in the *satf2583* (*rob*) gene of TF2758 and the predicted domain structure of its transcript. (A) Comparison of the *satf2580*-to-*satf2586* region with those of MW2, 8325-4, ATCC 49775, and TF2758. A part of the nucleotide sequence of each strain and the amino acid sequence are shown. The numbers shown on both sides indicate the nucleotide sequence and amino acid sequence positions in the ORF of *rob*. The nonsense codon created by the mutation (A to T) is indicated by an asterisk. (B) Structural characteristics of Rob. It contains a TetR_N superfamily domain within an AcrR domain.

**FIG 3:**
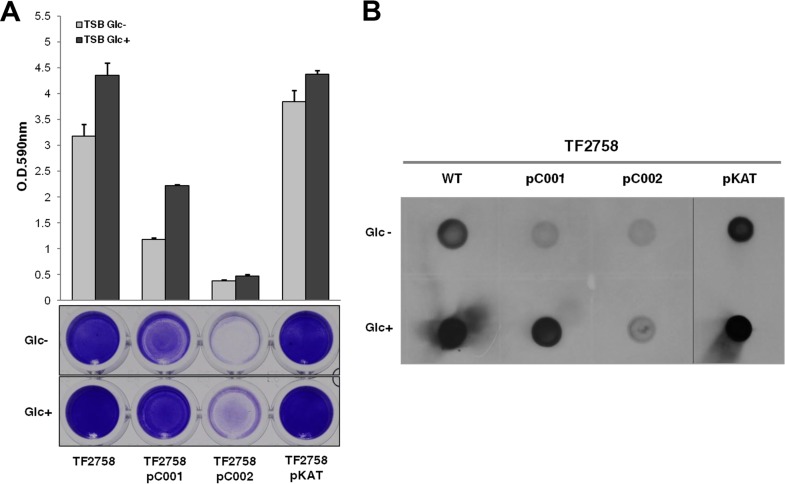
Rob and IcaR from FK300 reduce biofilm elaboration and PIA/PNAG synthesis in strain TF2758. Biofilm elaboration (A) and PIA/PNAG production (B) of TF2758 and TF2758 carrying pC001 (pKAT-*rob* [FK300]), pC002 (pKAT-*icaR* [FK300]), or pKAT. Bacteria were grown in TSB in the presence (Glc+) or absence (Glc-) of 1% glucose. Biofilm elaboration was measured using the polystyrene microtiter plate assay described in Materials and Methods. The averages and standard errors from each sample are shown. Extracts from overnight cultures were spotted on a membrane, and PIA/PNAG was detected by rabbit anti-PIA, as described in Materials and Methods. WT, wild type. Figures (WT, pC001, pC002, and pKAT) were compiled from two separate images on the same film.

As shown in Fig. 1, one of the clinically isolated strains, TF2758, showed a strong biofilm-elaborating ability and hyperproduction of PIA/PNAG (17). In order to elucidate the mechanism underlying the overproduction of biofilms in TF2758, a gene expression analysis using a custom microarray was performed. We used ATCC 49775 as a control because it was the strain most closely related to TF2758 by comparative genomic hybridization and a very-low-biofilm-elaborating strain (Fig. 1A and B). The results obtained showed that there were two strongly upregulated gene clusters: *satf2580* to *satf2586* (15- to 40-fold) and the *ica* operon (*satf2686* to *satf2689*; 2- to 10-fold) (Fig. 1C; see also Table S1 in the supplemental material). Sequencing of the TF2758 genome and comparisons with complete genomes of other *S. aureus* strains indicated that TF2758 possessed a nonsense mutation in the gene *satf2583* (Fig. 2A) and a missense mutation in the gene *icaR*, which resulted in the creation of a stop codon and an alteration in a nucleotide (A to T), respectively (Fig. S1). SATF2583 possessed regions homologous to the TetR family and AcrR family of transcriptional regulators, suggesting that it acts as a DNA-binding protein (Fig. 2B). In order to clarify the impact of SATF2583 on biofilm elaboration in *S. aureus*, we transformed TF2758 with the plasmid pC001, which is pKAT carrying open reading frame (ORF) SAOUHSC_2897 with a 5′-flanking region cloned from FK300, the *rsbU*-repaired derivative of NCTC8325-4. As shown in Fig. 3A, TF2758 carrying pC001 significantly lost its biofilm-elaborating ability, particularly in the absence of 1% glucose. PIA/PNAG detection by anti-PNAG revealed more clear-cut data (Fig. 3B). In the presence of 1% glucose, TF2758 carrying pC001 still retained its PIA/PNAG-producing ability; however, the amount produced was markedly smaller than that by the wild type. However, the production of PIA/PNAG was almost completely inhibited in the absence of 1% glucose. These results suggest that SATF2583 is a negative regulator of biofilm elaboration and also that the *satf2583* gene from FK300 is functional.

10.1128/mBio.02282-16.6 TABLE S1 List of genes upregulated in microarray experiments. Download TABLE S1, DOCX file, 0.02 MB. Copyright © 2017 Yu et al. 2017 Yu et al. This content is distributed under the terms of the Creative Commons Attribution 4.0 International license.

10.1128/mBio.02282-16.1 FIG S1  Identification of a missense mutation in the *icaR* gene of TF2758 and the domain structure of its transcript. (A) Comparison of the nucleotide sequence and amino acid sequence of the *icaR* gene among MW2, 8325-4, ATCC 49775, and TF2758. The numbers shown on both sides are the nucleotide sequence and amino acid sequence positions in the ORF of *icaR*. Amino acids (A to T) altered by the mutation at nucleotide position 103 (G to A) are indicated in red. (B) Structural characteristics of IcaR. It contains a TetR_N superfamily domain within an AcrR domain. Download FIG S1, TIF file, 0.2 MB. Copyright © 2017 Yu et al. 2017 Yu et al. This content is distributed under the terms of the Creative Commons Attribution 4.0 International license.

The *icaR* gene, located adjacent to the *ica* operon, is a member of the TetR family of transcriptional regulators (15, 18). IcaR was previously reported to repress *icaADBC* transcription by binding to a 42-bp region within the *ica* promoter (16). The missense mutation that we identified in *icaR* occurs in the helix-turn-helix (HTH) domain (Fig. S1 in the supplemental material) and may affect protein function. Therefore, we complemented TF2758 with pC002, which is pKAT carrying *icaR* from FK300. The resulting strain significantly decreased biofilm elaboration, and PIA/PNAG production was inhibited regardless of the presence or absence of glucose (Fig. 3A and B). These results suggest that *icaR* from FK300 is functional and that the *satf2583*-involved biofilm elaboration pathway occurs through and upstream of the *ica* operon. We tentatively named this ORF *rob* (*r*egulator *o*f *b*iofilm).

### Effects of Rob on biofilm elaboration, PIA/PNAG production, and *ica* operon expression in *S. aureus* FK300.

**FIG 4:**
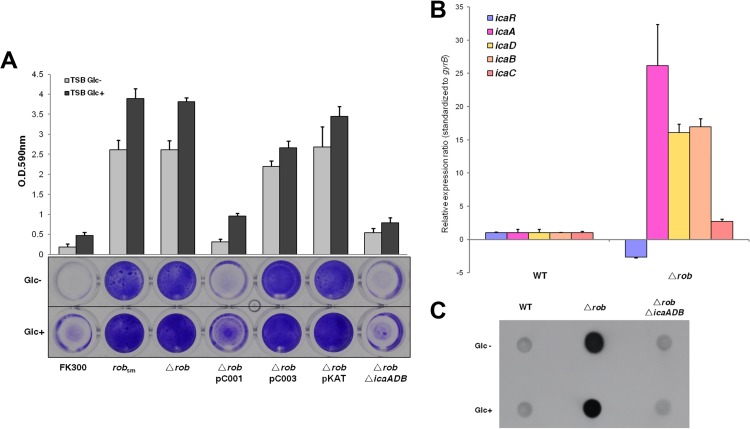
Effects of the *rob* deletion on biofilm elaboration and *ica* operon expression in FK300. (A) Biofilm elaboration in wild-type FK300 and its derivatives was assessed using the polystyrene microtiter plate assay described in Materials and Methods. The averages and standard errors from each sample are shown. sm, FK300 carrying a stop mutation at adenine nucleotide position 331 (A_331_ to T_331_); pC001, pKAT with *rob* (FK300); pC003, pKAT with *rob* (TF2758). (B) Quantitative measurements of *icaR* and *icaADBC* transcription by quantitative PCR. Total RNA preparation, cDNA synthesis, and then quantitative PCR were performed as described in Materials and Methods. Transcript levels in the *rob* deletion mutant compared to those in wild-type (WT) strain FK300 were assigned. The expression of the *gyrB* gene was used for sample normalization. Error bars indicate standard errors. (C) PIA/PNAG production was measured as described in the legend to Fig. 1.

TF2758 was resistant to transformation by the plasmids pKFT and pKOR1, which are used for allelic exchange in *S. aureus*. Since the *rob* gene in FK300 is functional, we selected strain FK300 for further studies on *rob* function. We introduced the same mutation found in TF2758 into *rob* in FK300 by allelic replacement. As shown in Fig. 4A, this mutation in *rob* resulted in a marked increase in biofilm elaboration in the presence or absence of 1% glucose. The deletion of the *rob* gene also increased biofilm elaboration in FK300. We complemented the *rob* deletion mutant with plasmids carrying the *rob* gene (SAOUHSC_2897) from FK300 (pC001) or the truncated *rob* gene (*satf2583*) from TF2758 (pC003). We found that the transformant of the *rob* deletion mutant with pC001 exhibited repressed biofilm elaboration, similarly to the control, FK300. In contrast, pC003 was unable to complement the *rob* deletion phenotype, which was similar to that of the *rob* deletion mutant carrying the mock vector pKAT (Fig. 4A).

In order to examine whether *rob* regulates biofilm elaboration through the *ica* operon, we measured *icaR* and *icaADBC* expression by reverse transcription-quantitative PCR (qRT-PCR) and PIA/PNAG production in wild-type and *rob* mutant strains of *S. aureus* FK300 (Fig. 4B). The results obtained indicated that the *rob* deletion mutant decreased *icaR* expression and increased *icaADBC* expression with a concomitant increase in PIA/PNAG production (Fig. 4C). The deletion of the *ica* operon in the FK300 *rob* deletion mutant abolished biofilm elaboration and PIA/PNAG production (Fig. 4A and C). Taken together, these results suggest that biofilm elaboration in the *rob* deletion mutant is *ica* dependent and that Rob, at least in part, represses *icaADBC* transcription.

### SAOUHSC_2898 (SATF2584) is involved in biofilm elaboration, which is under the control of Rob.

**FIG 5:**
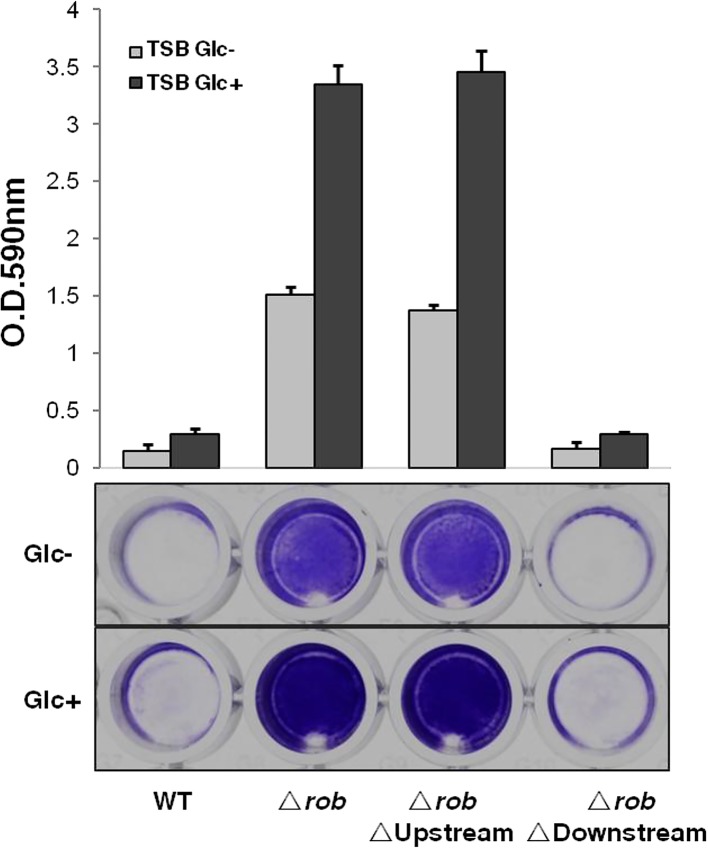
Biofilm elaboration in the *rob* deletion mutant requires a downstream gene(s) but not upstream genes. Bacteria were grown in TSB in the presence (Glc+) or absence (Glc-) of 1% glucose. Biofilm elaboration was measured using the polystyrene microtiter plate assay described in Materials and Methods. The averages and standard errors from each sample are shown. Δ*rob*, FK300 *rob* deletion mutant; Δ*rob*ΔUpstream, FK300 with deletions of *rob* and its upstream genes SAOUHC_ 2894, SAOUHC_ 2895, and SAOUHSC_2896; Δ*rob*ΔDownstream, FK300 with the deletion of *rob* and its downstream genes SAOUHSC_2898, SAOUHSC_2899, and SAOUHSC_2900. WT, wild type.

The results of a preliminary microarray analysis suggested that Rob suppresses the expression of the surrounding 7-gene cluster (*satf2580* to *satf2586*) and the *ica* operon in TF2758 (Fig. 1C; Table S1). Our RNA-sequencing (RNA-seq) data showed that these genes form operons (Fig. S2 in the supplemental material). Therefore, we hypothesized that Rob affects biofilm elaboration by repressing one or more genes in the *satf2580*-to-*satf2586* gene cluster. In order to test this possibility, we deleted upstream genes (SAOUHSC_2894 and SAOUHSC_2895) and downstream genes (SAOUHSC_2898, SAOUHSC_2899, and SAOUHSC_2900) in the FK300 *rob* deletion mutant. The results, shown in Fig. 5, revealed that the deletion of the upstream genes had no significant effect whereas the deletion of the downstream genes significantly reduced biofilm elaboration in the *rob* deletion mutant to a level similar to that of wild-type FK300.

10.1128/mBio.02282-16.2 FIG S2  Identification of *rob* operon and transcription start site of *rob* using RNA-seq analysis. (A) Visualization of RNA transcript identified by RNA-seq. Total RNA of FK300, FK300 Δ*rob*, and TF2758 was prepared from cultures grown for 6 h at 37°C. After removal of DNA contaminants and rRNA, libraries were generated and purified as described in Materials and Methods. RNA-seq reads were mapped to *S. aureus* NCTC8325. Genes with continuous coverage were considered to belong to the same operon. The ORFs of NCTC8325 are shown at the top of the figure. Transcripts identified by RNA-seq are represented as dashed arrows. The sequence from the predicted transcription start site (TSS) to the start codon of *rob* was shown at the bottom of the figure. (B) Diagrammatic representation of the *rob* promoter region. GENETYXMAC v.15 (Software Development Co., Ltd., Tokyo, Japan) was used for prediction of the −35, −10 sequence. The start codons of genes are indicated by arrows. The Rob-binding site is indicated by the open rectangle. The transcription start site of *rob* is highlighted by a bent arrow. Download FIG S2, TIF file, 1.5 MB. Copyright © 2017 Yu et al. 2017 Yu et al. This content is distributed under the terms of the Creative Commons Attribution 4.0 International license.

**FIG 6:**
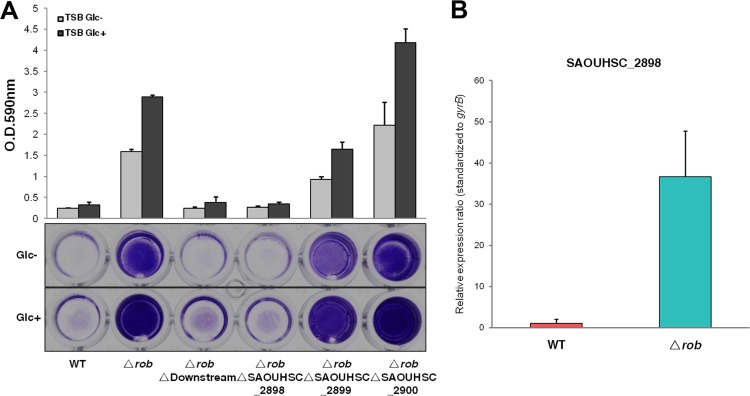
Contribution of SAOUHSC_2898 to biofilm elaboration by the *rob* deletion mutant and regulation of SAOUHSC_2898 expression by *rob* in FK300. Bacteria were grown in TSB in the presence (Glc+) or absence (Glc-) of 1% glucose. Biofilm elaboration was measured using the polystyrene microtiter plate assay described in Materials and Methods. (A) Effects of SAOUHSC_2898, SAOUHSC_2899, and SAOUHSC_2900 deletions on biofilm elaboration in the FK300 *rob* deletion mutant. The averages and standard errors from each sample are shown. (B) Transcription of SAOUHSC_2898 in the FK300 wild-type strain and its *rob* deletion mutant. Transcript levels in the *rob* deletion mutant compared to those in the wild-type strain were assigned. The expression of the *gyrB* gene was used for sample normalization. Error bars indicate standard errors. WT, wild type.

As described above, there are three adjacent genes located immediately downstream of *rob* that are under the control of one promoter, forming an operon. We deleted each gene individually in the FK300 *rob* deletion mutant (Fig. 6A). We found that only the SAOUHSC_2898 deletion caused a marked reduction in biofilm elaboration. The SAOUHSC_2899 deletion had a slight effect, whereas the SAOUHSC_2900 deletion had no effect on biofilm elaboration in the *rob* deletion mutant. Additionally, biofilm elaboration could be restored through complementation of the *rob* SAOUHSC_2898 double mutant with pC004, which carries the SAOUHSC_2898 gene from FK300 (Fig. S3 in the supplemental material). These results suggest that SAOUHSC_2898 is a critical factor mediating biofilm elaboration regulated by Rob.

10.1128/mBio.02282-16.3 FIG S3  Reduced biofilm elaboration in the *rob* SAOUHSC_2898 double mutant was restored through complementation with the SAOUHSC_2898 gene. Bacteria were grown in TSB in the presence (Glc+) or absence (Glc-) of 1% glucose. Biofilm elaboration was measured using the polystyrene microtiter plate assay described in Materials and Methods. The averages and standard errors from each sample are shown. pC004, pKAT with SAOUHSC_2898 (FK300). Download FIG S3, TIF file, 0.5 MB. Copyright © 2017 Yu et al. 2017 Yu et al. This content is distributed under the terms of the Creative Commons Attribution 4.0 International license.

In order to further confirm the regulation of SAOUHSC_2898 by *rob*, qRT-PCR was performed with RNA isolated from wild-type FK300 and the *rob* deletion mutant. The deletion of *rob* resulted in the increased expression of SAOUHSC_2898 (Fig. 6B). SAOUHSC_2898 is predicted to encode a 2-deoxy-d-gluconate 3-dehydrogenase that belongs to the oxidoreductase family (http://aureowiki.med.uni-greifswald.de/SAOUHSC_02898). Our results suggest that this enzyme is involved in some unknown biosynthetic pathway impacting biofilm elaboration. Rob may repress biofilm elaboration in FK300 by downregulating the transcription of the SAOUHSC_2898 gene.

### Rob recognizes a palindromic motif in its own promoter.

**FIG 7:**
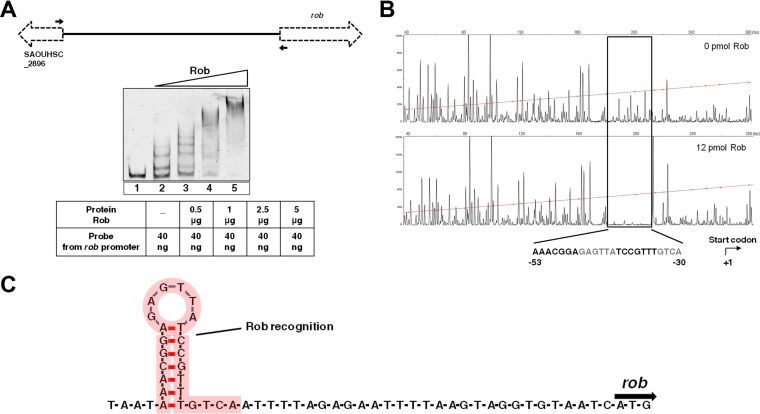
Rob binds to a palindromic motif in its own promoter. (A) EMSA for the DNA-binding activity of Rob to the intergenic region between SAOUHSC_2896 and *rob*. EMSA was performed in the absence (lane 1) or presence (lanes 2 to 5) of the Rob protein. The primers used to amplify the intergenic region for Rob binding are indicated by black arrows. (B) DNase I footprinting assay. The 6-FAM-labeled DNA probe was incubated with or without recombinant Rob (12 pmol) and then subjected to DNase I digestion. The rectangle indicates the region protected by Rob. The palindromic motif is shown in bold. (C) Schematic representation of the secondary structure of the binding region by Rob. The sequence bound by Rob is highlighted by red shading.

A microarray analysis showed that the inactivation of *rob* in TF2758 resulted in the increased expression of surrounding genes. Therefore, using electrophoretic mobility shift assay (EMSA) and DNase I footprint analyses, we investigated whether Rob directly binds to its own promoter and regulates this gene cluster’s transcription. We purified His-tagged Rob from *Escherichia coli* and analyzed its binding to an intergenic fragment between SAOUHSC_2896 and *rob* (Fig. 7A). Rob induced significant dose-dependent shifts in the probe’s mobility. A DNase I footprint analysis clearly demonstrated that Rob bound to a 24-nucleotide DNA (Fig. 7B). We then analyzed the secondary structure of the binding sequence. We found that it contained an almost perfect palindromic sequence (Fig. 7C). Interestingly, the transcription start site (TSS) of *rob* was predicted to be within the binding region of Rob by RNA-sequencing (RNA-seq) analysis (Fig. S2 in the supplemental material). These results suggest that Rob represses SAOUHSC_2898 transcription by recognizing the possible palindromic sequence present in the intergenic region of SAOUHSC_2896 and *rob*.

### Recombinant Rob binds to the *ica* promoter region.

**FIG 8:**
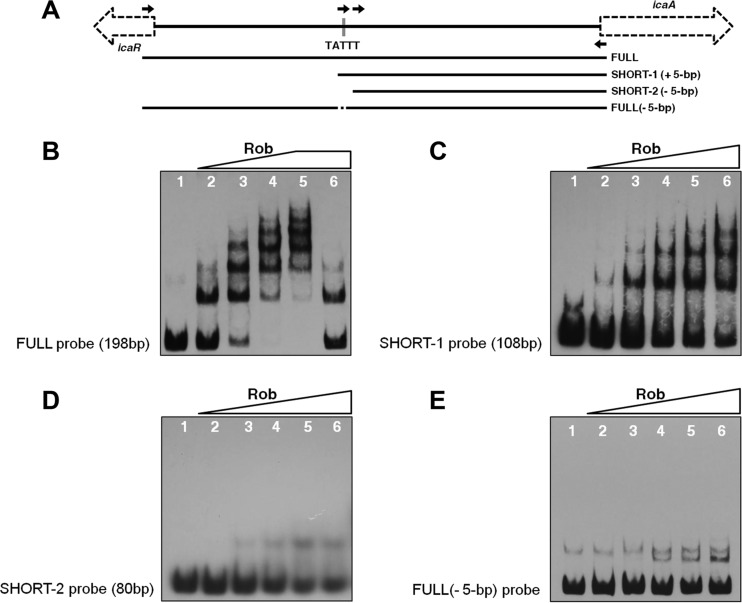
Rob binds to the *ica* promoter region and its binding is TATTT motif dependent. (A) Schematic representation of the design of DNA probes used in EMSAs. (B to E) EMSAs of Rob. Recombinant Rob was incubated with FULL (B), SHORT-1 (C), SHORT-2 (D), or FULL(−5-bp) (E) (2 ng/reaction mixture). The amounts (micrograms per reaction mixture) of Rob were as follows: lanes 1, 0; lanes 2 to 5, 0.1, 0.5, 1.0, and 1.5, respectively; lanes 6, 1.5 (with 100-fold excess of unlabeled specific competitor) (B) and 2.0 (C to E).

As shown in Fig. 4, the transcription levels of *icaADBC* were also significantly increased in the *rob* mutant. In order to investigate whether Rob directly modulates *icaADBC* expression by binding to the *ica* promoter, we used EMSA to analyze the Rob protein binding to a 198-bp probe (FULL) that contained the entire *icaR-icaA* intergenic region (Fig. 8A). As shown in Fig. 8B, the recombinant Rob protein induced several shifts, even with only 0.1 μg of FULL. Rob-DNA complex bands migrated in a ladder-like pattern with increases in Rob protein concentrations. Rob-DNA complexes were outcompeted with a 100-fold excess of unlabeled specific competitor DNA. These results suggest that Rob binds to the *icaR-icaA* intergenic region in a dose-dependent manner.

Jefferson et al. previously identified a 5-bp (TATTT) motif within the *icaR-icaA* intergenic region that controls the transcriptional regulation of the *ica* locus (16) (Fig. 8A). They suggested that an unknown repressor(s) utilize(s) the TATTT sequence in order to regulate *icaADBC* expression. Since Rob represses the *ica* transcription of the *ica* locus, we investigated whether Rob recognizes this 5-bp motif. We designed several additional probes for DNA-binding assays (Fig. 8A). A 108-bp probe (SHORT-1), the shortest oligonucleotide containing the 5-bp sequence lacking the 5′ 90-bp sequence of FULL, was dose dependently shifted by Rob (Fig. 8C). We then generated an oligonucleotide (SHORT-2) with a 28-bp deletion from the 5′ end of the SHORT-1 probe. As shown in Fig. 8D, SHORT-2 had no significant shift in the presence of Rob. In order to further investigate whether Rob recognizes the 5-bp motif, we made a 193-bp FULL(−5-bp) probe lacking the 5-bp TATTT sequence of FULL. As shown in Fig. 8E, FULL(−5-bp) was not shifted, as observed in FULL migration in Fig. 8B, suggesting that Rob was unable to bind to the 193-bp FULL(−5-bp) probe. Taken together, these results suggest that Rob recognizes and binds to the 5-bp motif within the *ica* promoter region.

**FIG 9:**
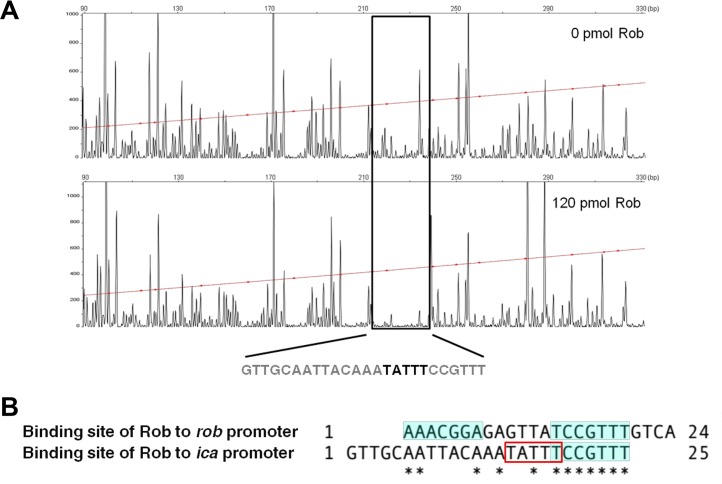
DNase I footprinting assay of Rob binding to the *ica* promoter region. (A) Footprint analysis of Rob binding to the *ica* promoter region. The sequence of the protected region is shown. (B) Comparison of the sites of Rob binding to the *rob* promoter region and *ica* promoter region. The palindromic sequence within the Rob-binding region is shaded. The bold letters (A) and rectangle (B) indicate the 5-bp TATTT motif.

We performed a DNase I footprint analysis to identify a Rob-binding site(s). As shown in Fig. 9, Rob protected a region of approximately 25 bp that included the 5-bp motif. In order to further confirm that the 5-bp motif is necessary for the binding of Rob to the *icaR*-*icaA* intergenic region, we attempted to screen for proteins bound to the *icaR-icaA* intergenic DNA fragment with or without the 5-bp motif using cytosolic proteins of FK300. The cell extract of the wild-type strain FK300 was mixed with magnetic beads conjugated with either the 198-bp FULL probe or 193-bp FULL(−5-bp) probe, and the bound proteins were then analyzed by matrix-assisted laser desorption ionization–time of flight mass spectrometry (MALDI-TOF MS). We found that Rob was present in proteins bound to the 198-bp probe but was absent in proteins bound to 193-bp FULL(−5-bp) (data not shown). Overall, these results strongly suggest that Rob recognizes and binds to the 5-bp TATTT motif within the promoter region of the *ica* locus.

## DISCUSSION

Biofilm elaboration is an important virulence determinant in certain types of *S. aureus* infections, particularly those involving implanted medical devices. Biofilm growth is influenced by a number of regulatory mechanisms. However, it is becoming increasingly apparent that the transcriptional regulation of biofilm-associated genes, such as *icaADBC*, is complex. Staphylococcal regulatory factors, including SarA, SigB, IcaR, TcaR, SrrAB, and Rbf, were previously shown to regulate *icaADBC* expression (11, 15, 19–22). In the present study, we identified a novel TetR/AcrR family regulator, Rob, which is a repressor of biofilm elaboration, by controlling SAOUHSC_2898, within a 7-gene cluster under the control of Rob. Furthermore, we demonstrated that Rob directly binds to the *icaR-icaA* intergenic region and represses *icaADBC*. The binding site in the *icaR-icaA* intergenic region contained the 5-bp motif, which has been suggested to control the transcriptional regulation of *icaADBC* (see Fig. S4 in the supplemental material) (16).

10.1128/mBio.02282-16.4 FIG S4  Diagrammatic representation of the *icaR-icaA* intergenic region. The start sites of *icaR* and *icaA* are indicated by arrows. The Rob-binding site is indicated by the open rectangle. The gray-shaded rectangle indicates the IcaR-binding site (16). The 5-bp TATTT motif, which has a functional role in the transcriptional regulation of the *ica* locus, is highlighted by a red frame (16). The Shine-Dalgarno sequence of *icaR* is underlined. The 5′ UTR of *icaR* is boxed (dashed line) in the sequence (31). The bent arrow indicates the transcriptional start site of *icaA* (45). Download FIG S4, TIF file, 0.2 MB. Copyright © 2017 Yu et al. 2017 Yu et al. This content is distributed under the terms of the Creative Commons Attribution 4.0 International license.

BLAST analysis showed that the *satf2580*-to-*satf2586* gene cluster, which was upregulated in the super-biofilm-elaborating strain TF2758, is also present in several other staphylococcal strains, but not in *Staphylococcus epidermidis*, which is among the best-studied and most clinically relevant biofilm-elaborating organisms (see Fig. S5 in the supplemental material). Therefore, a novel regulatory pathway appears to be involved in biofilm elaboration in *S. aureus*.

10.1128/mBio.02282-16.5 FIG S5  **C**omparison of the *satf2580*-to-*satf2586* region among different *Staphylococcus* species. The red frame represents the 7-gene cluster highlighted in this study. Download FIG S5, TIF file, 0.3 MB. Copyright © 2017 Yu et al. 2017 Yu et al. This content is distributed under the terms of the Creative Commons Attribution 4.0 International license.

SAOUHSC_2898 is predicted to encode a 2-deoxy-d-gluconate 3-dehydrogenase, which belongs to the oxidoreductase family. Oxidoreductases specifically act on the CH-OH group of donors with NAD^+^ or NADP^+^ as an acceptor. This enzyme participates in pentose and glucuronate interconversions, a metabolic pathway that has recently been shown to be significantly enriched in biofilm elaboration (23). An increase in the expression of oxidoreductase was previously reported to induce staphylococcal biofilm elaboration (24). The detailed characterization of SAOUHC_2898 will provide an insight into *ica*-dependent biofilm elaboration.

**FIG 10:**
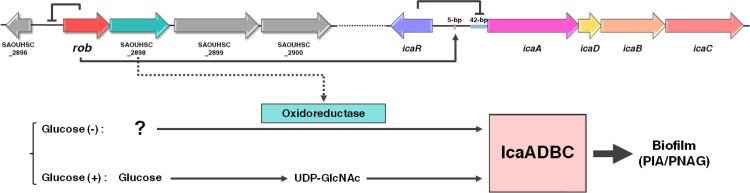
Proposed model for regulation of PIA/PNAG synthesis by Rob in *S. aureus* FK300. The *rob* gene product represses the expression of the surrounding 7-gene cluster, including *rob* and SAOUHSC_2898. The gene product of SAOUHSC_2898 may function as an oxidoreductase in a hypothetical pathway through which glucose-independent, *icaADBC*-dependent polysaccharide accumulation occurs. *rob* also recognizes the TATTT motif in the *ica* promoter region and binds to this region. The binding of Rob to the *ica* promoter region may suppress the expression of the *icaADBC* locus. 5-bp, the TATTT motif important for the expression of *ica* locus; 42-bp, the IcaR-binding region. Arrows correspond to activation, and bars correspond to repression.

SAOUHSC_2897 and SAOUHSC_2898 were previously reported to be glucose induced biofilm accessory genes of an operon designated *gbaAB* (25). However, in our assay, the complementation of TF2758 with SAOUHSC_2897 (*rob*) completely canceled PIA/PNAG production in the absence of 1% glucose. Furthermore, the addition of glucose did not alter the amount of PIA/PNAG produced by the FK300 *rob* deletion mutant (Fig. 4C). Thus, it is reasonable to assume that an SAOUHSC_2898-catalyzed pathway controlled by SAOUHSC_2897 (*rob*) affects biofilm elaboration in a glucose-independent manner (Fig. 10).

A number of regulators, including SigB, SarA, and SarX, and two-component signal transduction systems (TCSs) have been shown to affect staphylococcal biofilm elaboration (26–29). Our genetic analyses showed that the deletion of *ica* genes or SAOUHSC_2898 resulted in a loss in the ability to elaborate biofilms in the FK300 *rob* mutant. The *rob* mutant showed decreased *icaR* expression and increased *icaADBC* transcription, suggesting that *rob* regulates an *ica*-dependent pathway for biofilm elaboration, at least in part by activating *icaR* expression.

Some factors regulate *icaADBC* expression by binding to the *icaR-icaA* intergenic region (15, 16, 22, 28). Although Rob is one of the TetR/AcrR family regulators in *S. aureus*, its role in the regulation of biofilms is not completely clear. Jefferson et al. previously reported that the TATTT sequence has a functional role in the transcriptional regulation of the *ica* locus (16). The simple deletion of the TATTT motif in *S. aureus* MN8m markedly increased biofilm elaboration and the transcription of *icaADBC*. Jefferson et al. (16) hypothesized the presence of an uncharacterized repressor(s) recognizing and binding to the motif. Most recently, Schwartbeck et al. also showed that the *S. aureus* isolates carrying the 5-bp deletion exhibited a mucoid phenotype and strong biofilm formation (30). These mucoid isolates were protected against phagocytosis and survived better under starvation conditions. The results of the present study demonstrated that Rob binds to an *icaR*-*icaA* intergenic region of approximately 25 bp, including the 5-bp TATTT motif, strongly suggesting that Rob is the postulated repressor reported by Jefferson et al., and further support the idea of *rob* regulating biofilm elaboration in an *ica*-dependent manner. A comparison of the Rob-binding site in the *icaR-icaA* intergenic region with that in the *rob* promoter revealed that the right half of the palindrome sequence was also present in the *icaR-icaA* intergenic region (Fig. 9). This palindrome-like sequence may be recognized by Rob. A previous study showed that the TATTT motif has a functional role in the transcriptional regulation of the *ica* locus but not *icaR* transcription (16). Ruiz de los Mozos et al. (31) recently demonstrated that the 5′ and 3′ untranslated region (UTR) base pairings of *icaR* mRNA control its transcription in *S. aureus*. The 5-bp motif is located within the 5′ UTR of *icaR* (Fig. S4 in the supplemental material). The possibility of an interaction between Rob and 5′ UTR to control base pairing remains elusive.

Taken together, the results of the present study suggest that Rob controls the two different pathways of biofilm elaboration in *S. aureus*. The TetR-family transcriptional regulator Rob affects biofilm elaboration through SAOUHSC_2898 and by recognizing/binding the TATTT motif in an *ica*-dependent manner. These results provide additional insights into the transcriptional regulation of the *ica* locus. Both Rob-mediated pathways will be investigated in more detail in future studies.

## MATERIALS AND METHODS

### Bacterial strains and growth media.

The bacterial strains and plasmids used in the present study are listed in Table 1. The *S. aureus* strain designated TF2758 is a clinical isolate from an atheroma in Japan. *S. aureus* ATCC 49775 served as a negative-control, non-biofilm-producing strain. *S. aureus* FK300, an *rsbU*-repaired derivative of strain NCTC8325-4, was used in a functional study of the role of *rob*. *S. aureus* RN4220 (32) was used as the initial recipient for the manipulation of recombinant plasmids. *S. aureus* was routinely grown in tryptic soy broth (TSB; Becton, Dickinson Microbiology Systems, Cockeysville, MD) or on tryptic soy agar (TSA) plates. Tetracycline (Tc; 5 μg/ml) or chloramphenicol (Cp; 10 μg/ml) was added as necessary. *Escherichia coli* strain DH5α was used for the construction and maintenance of plasmids. *E. coli* was grown in lysogeny broth (LB) (5 g yeast extract, 10 g polypeptone, and 10 g NaCl per liter; pH 7.2) or on LB agar. When required, ampicillin (Ap; 100 μg/ml), kanamycin (Kn; 30 μg/ml), Tc (10 μg/ml), or Cp (10 μg/ml) was added to the culture medium.

**TABLE 1:** Strains and plasmids used in the present study

Strain or plasmid	Relevant characteristic(s)	Source or reference
Strains		
*S. aureus*		
TF2758	Wild-type clinical isolate, biofilm positive	This study
ATCC 49775	Wild-type clinical isolate, biofilm negative	ATCC
FK300	Derivative of NCTC8325-4 (*rsbU* repaired)	Laboratory stock
RN4220	Restriction-negative strain, NCTC8325-4 derivative	32
TF2758 pC001	TF2758 complemented with pC001	This study
TF2758 pC002	TF2758 complemented with pC002	This study
*rob*_*sm*_ strain	FK300 with a stop mutation in *rob*	This study
Δ*rob* strain	FK300 Δ*rob*	This study
Δ*rob* pC001 strain	FK300 Δ*rob* complemented with pC001	This study
Δ*rob* pC003 strain	FK300 Δ*rob* complemented with pC003	This study
Δ*rob* pKAT strain	FK300 Δ*rob* complemented with pKAT	This study
Δ*rob* Δ*icaADB* strain	FK300 Δ*rob* Δ*icaADB*	This study
Δ*rob* ΔUpstream strain	FK300 Δ*rob* ΔSAOUHSC_2894 ΔSAOUHSC_2895 ΔSAOUHSC_2896	This study
Δ*rob* ΔDownstream strain	FK300 Δ*rob* ΔSAOUHSC_2898 ΔSAOUHSC_2899 ΔSAOUHSC_2900	This study
Δ*rob* ΔSAOUHSC_2898 strain	FK300 Δ*rob* ΔSAOUHSC_2898	This study
Δ*rob* ΔSAOUHSC_2899 strain	FK300 Δ*rob* ΔSAOUHSC_2899	This study
Δ*rob* ΔSAOUHSC_2900 strain	FK300 Δ*rob* ΔSAOUHSC_2900	This study
*E. coli*		
DH5α	Cloning strain	TaKaRa
BL21(DE3)	Host for recombinant protein production	Novagen
Plasmids		
pGEM-T Easy	Cloning vector	Promega
pKAT	*E. coli-S. aureus* shuttle vector	36
pC001	Vector for complementation experiments; containing *rob* from FK300 cloned in pKAT	This study
pC002	Vector for complementation experiments; containing *icaR* from FK300 cloned in pKAT	This study
pC003	Vector for complementation experiments; containing *rob* from TF2758 cloned in pKAT	This study
pC004	Vector for complementation experiments; containing SAOUHSC_2898 from FK300 cloned in pKAT	This study
pKFT	Vector for allele replacement	34
pET-28a(+)	*E. coli* expression plasmid	Novagen
pET-22b(+)	*E. coli* expression plasmid	Novagen
pET28a-*rob*	His-Rob expression plasmid	This study
pET22b-*icaR*	His-IcaR expression plasmid	This study

### Plasmid and strain construction.

Routine DNA manipulations were performed as previously described (33). FK300 mutants were constructed by allele replacement using pKFT (34). PCR was performed using KOD-Plus-Neo (Toyobo, Japan) under appropriate cycling conditions. The oligonucleotides used in this study are listed in Table 2. Fragments were cloned into the plasmid pKFT using restriction enzymes and transformed into *E. coli* DH5α. Recombinant plasmids were then introduced into DNA restriction system-deficient *S. aureus* RN4220 by electroporation (35). Modified plasmids were electroporated into *S. aureus* FK300 for allele replacement. Markerless deletion mutants were screened by PCR from tetracycline-sensitive colonies. Fragments were confirmed by DNA sequencing using the BigDye Terminator v 3.1 cycle sequencing kit (Applied Biosystems, USA).

**TABLE 2:** Primers used in the present study

Purpose and primer	Sequence (5′ to 3′)
Plasmid and strain construction	
*rob*_*sm*_-1	ACAACGCCCTTAATTGTTGCC
*rob*_*sm*_-2	GCAACAATTAAGGGCGTTGTTACCAAAG
*rob*-1	TACCAAGCTTCCTCTAACAACTGTTTTAC
*rob*-2	CATCAACTAGTTTGTGCGCTATTTCTTC
*rob*-3	GCTGTTGCAATCATTATCAACTAGTG
*rob*-4	AGGTAAAGCTTTAGCGTATTGTAGCG
*rob*Up-1	AACTAAGCTTTGCCATCGTACTACTAG
*rob*Up-2	GAGCAAAGACGCATCACAGCGGTCTGCTAAAATGAAATTC
*rob*Up-3	GAATTTCATTTTAGCAGACCGCTGTGATGCGTCTTTGCTC
*rob*Up-4	CGGCAAGCTTAATGAGGATATCAAGACG
*rob*Down-1	AACTAAGCTTATCACTCAGATCACCTTC
*rob*Down-2	GCGGAATCAGGGAGTGGTTCGTGCGCTATTTCTTCAATTC
*rob*Down-3	GAATTGAAGAAATAGCGCACGAACCACTCCCTGATTCCGC
*rob*Down-4	GTAAACAAAAATAAGCTTGGTCAGCC
SAOUHSC_2898-1	AACTAAGCTTATCACTCAGATCACCTTC
SAOUHSC_2898-2	GGCTTGATTCCTTCAGAAACGTGCGCTATTTCTTCAATTC
SAOUHSC_2898-3	GAATTGAAGAAATAGCGCACGTTTCTGAAGGAATCAAGCC
SAOUHSC_2898-4	GCGAATAAAGCTTCATCCATACG
SAOUHSC_2899-1	GCCGTCTTGGGATCCTCATTAAC
SAOUHSC_2899-2	GGATAATCAGCAGCATAAAGCGGTACACCTTTAGGATCTG
SAOUHSC_2899-3	CAGATCCTAAAGGTGTACCGCTTTATGCTGCTGATTATCC
SAOUHSC_2899-4	CTATGGATCCTTCTTCAGTATC
SAOUHSC_2900-1	TTAGGATCCAAAGGTGCGCTCATTATG
SAOUHSC_2900-2	GAATATAACCTAAGTGACCGCCAGGAATAAAGATGAGCAC
SAOUHSC_2900-3	GTGCTCATCTTTATTCCTGGCGGTCACTTAGGTTATATTC
SAOUHSC_2900-4	CTATTTTGGATCCGTTTACAAC
*icaR*-1	TGGTGAAGCTTGATCAACGATAGTATC
*icaR*-4	TAATAAAGCTTGATACCATCGTACTC
*ica*-1	AATTGGATCCTCATTGAACAAGAAGCC
*ica*-2	TAATACTAGTTGTCCCCCTTGAGCCCATC
*ica*-3	GATGAAACTAGTTATGAAAATGCTTATCC
*ica*-4	AATTGTAACACTAAGGATCCACCCTCC
SAOUHSC_2898-comF	AGGTGGATCCTTCGAAATGTGCTTGC
SAOUHSC_2898-comR	ACATAAGCTTGATCTACCAAGGC
Quantitative PCR	
*gyrB* for	AGGTCTTGGAGAAATGAATG
*gyrB* rev	CAAATGTTTGGTCCGCTT
*icaR* for	CGCCTGAGGAATTTTCTG
*icaR* rev	GGATGCTTTCAAATACCAAC
*icaA* for	AGTTGTCGACGTTGGCTAC
*icaA* rev	CCAAAGACCTCCCAATGT
*icaD* for	ACCCAACGCTAAAATCATCG
*icaD* rev	GCGAAAATGCCCATAGTTTC
*icaB* for	ATACCGGCAACTGGGTTTAT
*icaB* rev	TGCAAATCGTGGGTATGTGT
*icaC* for	CTTGGGTATTTGCACGCATT
*icaC* rev	GCAATATCATGCCGACACCT
SAOUHSC_2898 for	ATTGACACCTCGTGACGTTG
SAOUHSC_2898 rev	CCACTTGATACGTTGACGAC
EMSA and DNase I footprint analysis	
*ica*-p-F	ATTGCGTTATCAATAATCTTATCCTTC
*ica*-p-R (5-biotin)	TTGCAATTCCTTTACCTACCTTTC
*ica*-p-R′	TTGCAATTCCTTTACCTACCTTTC
*ica*-p-F-s1	ACAAATATTTCCGTTTAATTATAACAAC
*ica*-p-F-s2	AATCTATTGCAAATTAAAATACTATC
5bp-deletion-2	TTGTTGTTATAATTAAACGGTTTGTAATTGCAACTTAATT
5bp-deletion-3	AATTAAGTTGCAATTACAAACCGTTTAATTATAACAACAA
*rob*-p-F	CGTCTTTGCTCTCTAGTTAAAGAC
*rob*-p-R	CTATTCTCTTTTGCATCTTTTCGC
T7 promoter-1 Cy3^a^	TAATACGACTCACTATAGGG
Fp-M13-F	GTTTTCCCAGTCACGAC
Fp-M13-R 6-FAM^b^	CAGGAAACAGCTATGAC
pET-28a-Rob-F	AGGTGGATCCATGCGAAAAGATGC
pET-28a-Rob-R	TAACAAGCTTTTAGTCATTACGTCCCACC
pET-22b-IcaR-F	GGAATTCCATATGCACCACCACCACCACCACTTGAAGGATAAGATTATTGATAACGC
pET-22b-IcaR-R	CCCAAGCTTTTATTTCTTCAAAAATATATTTAGTAGCG

a Cy3 labeled at the 5′ end.

b 6-FAM labeled at the 5′ end.

In complementation experiments, genes were amplified by PCR using the corresponding primer pairs and then cloned into the HindIII site of pKAT (36). The plasmids pC001, pC002, and pC003 carrying *rob*-FK300, *icaR*-FK300, and *rob*-TF2758 genes, respectively, were constructed and transformed into the *S. aureus* strains listed in Table 1 by electroporation. The inserts in all plasmid constructs were verified by PCR and DNA sequencing.

### Biofilm assay.

A biofilm assay using polystyrene plates was performed as described previously (37) with a few modifications. In brief, overnight cultures were diluted 1:100 with TSB. Ten microliters of this dilution was then transferred, in triplicate, into flat-bottom 96-well polystyrene plates (TrueLine; Nippon Genetics Co., Ltd., Japan) containing TSB or TSB plus 1% glucose. After incubation at 37°C for 24 h, the wells were gently washed three times with 300 μl of sterile phosphate-buffered saline (PBS) (137 mM NaCl, 2.7 mM KCl, 10 mM Na_2_HPO_4_⋅12H_2_O, and 1.8 mM KH_2_PO_4_, pH 7.4), and the biofilm was stained with 1% crystal violet for 15 min. Unbound crystal violet was then removed by washing the plate in a container by immersing and agitating it gently 10 times in tap water. Biofilm-bound crystal violet was solubilized in 200 μl of 33% glacial acetic acid at room temperature for 15 min. The extracts were diluted 10-fold, and absorbance at 590 nm was measured with an Immuno-Mini NJ-2300 spectrophotometer (Nalge Nunc International K.K., Tokyo, Japan).

### PIA/PNAG detection.

The ability of *S. aureus* strains to produce PIA/PNAG was tested according to a previously described protocol (9). Briefly, *S. aureus* strains were grown at 37°C overnight with shaking in 3 ml of TSB. Cultures were then diluted 1:1,000 in the appropriate medium, and 4 ml of this cell suspension was used to inoculate sterile 12-well polystyrene plates (TrueLine; Nippon Genetics Co., Ltd., Japan). After a 24-h static incubation at 37°C, the cells were resuspended in 50 μl of 0.5 M EDTA (pH 8.0) and incubated for 5 min at 100°C. Cells were removed by centrifugation, and 40 μl of the supernatant was incubated with 10 μl of proteinase K (20 mg/ml; Nacalai Tesque, Kyoto, Japan) at 37°C for 30 min. After the addition of 10 μl of Tris-buffered saline (20 mM Tris-HCl, 150 mM NaCl [pH 7.4]) containing 0.01% bromophenol blue, 5 μl was immobilized on a nitrocellulose membrane (Amersham Protran NC 0.45; GE Healthcare, Buckinghamshire, United Kingdom) and dried at room temperature. The membrane was blocked with 5% skimmed milk in PBS with 0.1% Tween 20, and this was followed by a 2-h incubation with rabbit anti-PNAG antiserum (38) diluted at 1:10,000. Bound antibodies were detected with peroxidase-conjugated goat anti-rabbit immunoglobulin G (IgG) antibodies (MP Biomedicals, LLC-Cappel Products, Ohio, USA) diluted 1:10,000 and developed with Pierce enhanced chemiluminescence (ECL) Western blotting substrate (Thermo Scientific, Rockford, IL).

### RNA isolation, RT, and real-time PCR.

Overnight *S. aureus* cultures were diluted in fresh TSB containing 1% glucose to an initial optical density (OD) of 0.02 at 660 nm and harvested after a 6-h incubation with shaking at 37°C. Total RNA was isolated using the FastRNA Pro Blue kit (MP Biomedicals, Santa Ana, CA, USA) according to the manufacturer’s instructions. DNA was removed by a treatment with RQ1 RNase-free DNase (Promega, Madison, WI) at 37°C for 30 min. After inactivation of DNase, PCR was performed to confirm the absence of contaminating DNA. RNA was then reverse transcribed with a Transcriptor First-Strand cDNA synthesis kit (Roche, Mannheim, Germany). The resulting cDNA was diluted 10-fold with Tris-EDTA (TE) buffer (10 mM Tris-HCl, 1 mM EDTA, pH 8.0) and then used as a template in the real-time PCR reaction. Quantitative real-time reverse transcription-PCR (qRT-PCR) was performed with the SsoAdvanced Universal SYBR green SuperMix (Bio-Rad, Hercules, CA) using a CFX96 real-time PCR detection system (Bio-Rad). The thermal cycling conditions used were as follows: 95°C for 1 min, followed by 40 cycles of 95°C for 15 s, 60°C (*icaR* and *icaA*) or 62°C (*gyrB* and SAOUHSC_2898) for 15 s, and 72°C for 30 s. All PCR runs were performed in triplicate, and data were analyzed using the CFX Manager software (version 3.0; Bio-Rad) according to the manufacturer’s instructions. The housekeeping gene gyrase subunit B (*gyrB*) was used as a reference gene to normalize the expression level of the target gene in each reaction. Real-time PCR primers are listed in Table 2.

### Microarray analysis.

The design and preparation of probes, which cover more than 98% of the open reading frames (ORFs) of *S. aureus* MW2, and their immobilization on the glass slide were described elsewhere (39). RNA extraction (after a 2-h incubation) and cDNA synthesis were performed as described above. cDNA was fluorescently labeled with Alexa Fluor 555 (Cy3) and Alexa Fluor 647 (Cy5) (Thermo Fisher Scientific, Oregon, USA). Labeled cDNA samples were mixed and hybridized to the slides. After washing, fluorescent signals were detected using a GenePix 4000B microarray scanner (Axon Instruments). Data were then normalized and analyzed using Array Vision 8.0 software (Imaging Research, CT, USA). The non-biofilm-elaborating strain ATCC 49775, the genotype of which is the most closely related to TF2758 in our Japanese clinical isolate collection, was used as the reference strain.

### Transcriptomic analysis of *rob* operon via RNA-seq.

Overnight *S. aureus* FK300 (wild-type, Δ*rob*) and TF2758 cultures were diluted in fresh TSB to an initial density of 0.02 at 660 nm and harvested after a 6-h incubation with shaking at 37°C. Total RNA was isolated using the FastRNA Pro Blue kit (MP Biomedicals, Santa Ana, CA, USA) according to the manufacturer’s instructions. To eliminate DNA contamination, 1 µg total RNA in each sample was treated with 3 µl (1 U/µl) of RQ1 RNase-free DNase (Promega, Madison, WI) at 37°C for 30 min. After digestion by DNase, PCR of the *gyrB* gene was performed to confirm the absence of contaminating DNA. The concentration and quality of total RNA were determined using a Qubit 2.0 fluorometer (Thermo Fisher Scientific) and Agilent 2200 TapeStation (Agilent Techonogies), respectively. rRNA was removed using the Ribo-Zero bacterial kit (Epicentre). Removal of rRNA was confirmed by the Agilent 2200 TapeStation.

Libraries were generated using the ScriptSeq v2 RNA-Seq (Epicentre) and purified using the Minelute PCR purification kit (Qiagen) according to the manufacturer’s instructions. Libraries were sequenced using the index sequences of TruSeq v2 LT sample preparation kit on the Illumina MiSeq platform. Sequence reads were preprocessed for quality, trimmed, and mapped to *S. aureus* strain NCTC8325 (GenBank accession number NC_007795) as the reference genome using CLC Genomics Workbench software platform ver. 9 (Qiagen) and Integrative Genomics Viewer (IGV) ver. 2.

### Sequencing of the TF2758 genome.

Genomic DNA was extracted using the lysostaphin and QIAamp DNA minikit (Qiagen, Germany) according to the manufacturer’s instructions. Libraries were prepared for sequencing with Nextera DNA kits (Illumina, USA) and were sequenced with the Illumina GAIIx system according to Illumina protocols. The raw reads were trimmed and assembled using a SOAPdenovo assembler. The draft genome sequence was automatically annotated using the Microbial Genome Annotation Pipeline (MiGAP) (40) and was manually curated using IMC-GE software (In Silico Biology, Inc., Kanagawa, Japan).

### Protein purification.

To elucidate the DNA-binding properties of Rob, the full-length open reading frame (ORF) of *rob* was amplified from FK300 genomic DNA using primers pET-28a-Rob-F/pET-28a-Rob-R (Table 2) and cloned into the expression vector pET-28a(+) (Novagen) to obtain pET28a-*rob*. The plasmid was then transformed into *E. coli* BL21(DE3), and bacteria were grown at 37°C in 300 ml LB containing 30 μg/ml kanamycin to an optical density (OD) of 0.5 at 600 nm. Expression of Rob was induced with 0.5 mM IPTG (isopropyl-β-d-thiogalactopyranoside; Nacalai Tesque, Kyoto, Japan) and with incubation at 37°C for another 6 h. Cells were harvested by centrifugation and frozen at −80°C. Cell pellets were thawed in lysis buffer (50 mM NaH_2_PO_4_ and 300 mM NaCl, pH 8.0) and lysed by sonication on ice. Cell debris was removed by centrifugation (10,000 × *g* at 4°C for 20 min), and the supernatant was used for isolation of His_6_-tagged Rob fusion protein by using Talon metal affinity resins (Clontech Laboratories, Inc.) according to the company’s protocol. The expression and purity of the protein were analyzed by sodium dodecyl sulfate-polyacrylamide gel electrophoresis (SDS-PAGE) using a 12% polyacrylamide gel. Protein concentrations were measured using the Bio-Rad protein assay (Bio-Rad, Hercules, CA) with bovine serum albumin (BSA) as the standard protein. The recombinant His-tagged IcaR protein was purified as described elsewhere (18).

### Electrophoretic mobility shift assays (EMSAs).

Gel shift assays were performed as described previously (16) with the following modifications. DNA fragments corresponding to the *icaR-icaA* intergenic region and promoter region of *rob* were amplified by PCR with the primers listed in Table 2. PCR products were purified using the QIAquick gel extraction kit (Qiagen). A 20-μl binding reaction mixture containing 0.1 to 2 μg of purified recombinant protein and 1 μg of sonicated salmon sperm DNA as well as 1 μg of poly(dI-dC) in binding buffer (10 mM HEPES [pH 8.0], 60 mM KCl, 4 mM MgCl_2_, 0.1 mM EDTA [pH 8.0], 0.1 mg/ml BSA, 0.25 mM dithiothreitol [DTT], and 5% glycerol) was incubated at room temperature for 15 min before the addition of 2 μg of the biotin-labeled probe. The reaction mixtures were incubated for an additional 20 min and then electrophoresed in a 5% polyacrylamide gel in prechilled 1× Tris-borate-EDTA (TBE) buffer. DNA was then transferred onto a nylon membrane (Biodyne B; Pall, USA), and band shifts were detected by exposing dried membranes to X-ray films. In order to measure the binding of Rob to its promoter region, a gel shift assay was performed using an alternative method as described previously (41).

### DNase I footprint analysis.

Footprinting was performed according to a previously described method (42). DNA fragments were generated by PCR with TaKaRa LA *Taq* (TaKaRa Bio Inc., Shiga, Japan). PCR products were purified and ligated with pGEM-T Easy (Promega) using Ligation High ver. 2 (Toyobo, Osaka, Japan). The resulting plasmids were then used as a template for the amplification of DNA probes using the primer pair Fp-M13-F and Fp-M13-R (5′ 6-carboxyfluorescein [FAM] labeled). DNA fragments (0.45 pmol) were mixed with purified proteins in 50 μl of a reaction mixture containing the same buffer used for gel shift assays. After a 20-min incubation at room temperature, the reaction mixtures were treated with 0.3 U of DNase I (Promega, Madison, WI) for 1 min and then purified by phenol-chloroform-isoamyl alcohol (CIAA) extraction and ethanol precipitation. After purification, the samples were analyzed using an ABI 3130xl Genetic Analyzer equipped with the Peak Scanner software (Applied Biosystems).

### Isolation and identification of proteins binding to the *ica* promoter.

A cell extract was isolated from strain FK300 as previously described with some modifications (43). Briefly, cultured *S. aureus* cells were pelleted and then washed with buffer A (20 mM Tri-HCl, 5 mM MgCl_2_, 0.1 M EDTA, and 5% glycerol, pH 7.8). Cell pellets were resuspended in 10 ml of buffer A and treated with lysostaphin (0.1 mg/ml) at 4°C for 1 h. After freezing at −80°C and thawing at 4°C twice, 6 ml of buffer A (containing KCl at a final concentration of 1.3 M) was added and incubated on ice for 40 min. The cell lysate was treated with DNase I (10 μg/ml) and RNase A (10 μg/ml) at room temperature for 30 min. After centrifugation for 30 min at 40,000 × *g*, the supernatant was dialyzed against distilled water overnight and stored at −80°C.

Biotinylated DNA was prepared as described above. DNA was immobilized on 2 mg of streptavidin-coated magnetic beads (Dynabeads M-280 streptavidin; Life Technologies, Inc.) according to the manufacturer’s protocol. After washing, 100 μl of the cell extract was added and incubated at room temperature for 30 min in gel shift binding buffer. The beads were washed twice with buffer B (10 mM HEPES [pH 8.0], 60 mM KCl, 4 mM MgCl_2_, 1 mM EDTA [pH 8.0], 1 mM DTT, and 5% glycerol) containing 0.5 μg/ml of salmon sperm DNA and then washed twice with buffer B. The bound proteins were eluted from immobilized DNA with buffer B containing 0.5 M NaCl. The eluates from two binding reactions were pooled and concentrated by methanol-chloroform precipitation. Proteins were separated by SDS-PAGE, followed by Coomassie blue or silver staining. Prior to in-gel trypsin digestion, excised gel pieces were destained and submitted to reduction with DTT and alkylation with iodoacetamide as described previously (44). After being dried, the gel pieces were subjected to trypsin digestion at 35°C overnight with XL-TrypKit (APRO Sci, Japan). Digested peptides were transferred to new tubes and evaporated to <10 μl in a vacuum centrifuge evaporator, and this was followed by liquid chromatography-tandem mass spectrometry (LC-MS/MS) analyses for protein identification. LC-MS/MS analyses were performed on a nanoflow liquid chromatograph coupled with nano-electrospray MS, a Triple TOF 5600 system (AB Sciex, Concord, Ontario, Canada) equipped with an Eksigent cHiPLC-nanoflex system (AB Sciex). The nano-high-performance liquid chromatography (HPLC) columns used were the cHiPLC trap column (200 μm by 0.5 mm; ChromXP C_18_-CL; 3 μm) and nano-cHiPLC analytical capillary column (75 μm by 15 cm; ChromXP C_18_-CL; 3 μm, 120 Å). Tryptic peptides (2 μl) were loaded, and trapping and desalting were performed at 2 μl/min for 10 min with 0.1% formic acid. The trapped peptides were separated by a linear gradient at a flow rate of 0.3 μl/min, followed by their introduction into the source of the mass spectrometer online. Mobile phase A (0.1% formic acid in H_2_O) and mobile phase B (0.1% formic acid in acetonitrile) were used to establish a 45-min gradient comprising 25 min of 2 to 32% B, 1 min of 32 to 90% B, 4 min of 90% B, and a final decrease to 2% B, which was followed by reequilibration at 2% B for 15 min. Eluted peptides from the column were analyzed with a Triple TOF 5600 using an ion spray voltage of 2.2 kV. Product ions were scanned in a mass range from 230 *m/z* up to 1,500 *m/z*. MS/MS data acquisition was performed using Analyst 1.5.2 (AB Sciex), and proteins were identified by means of an automated database search using ProteinPilot software (AB Sciex).
