# Supplementary material for: A Novel Repressor of the ica Locus Discovered in Clinically Isolated Super-Biofilm-Elaborating Staphylococcus aureus
Source: mBio. 2017 Jan 31;8(1):e02282-16. doi: 10.1128/mBio.02282-16 (PMC5285506; doi:10.1128/mBio.02282-16)
Supplement: TABLE S1 [file mbo001173169st1.docx]

**TABLE S1** List of genes up-regulated in microarray experiments

| MW2 (TF2758)  open reading frame | Name | Product | Fold*^a^* | | |
| --- | --- | --- | --- | --- | --- |
| MW2495 (*satf2580*) |  | Glyoxalase protein | 40.83 | 16.41 | 25.94 |
| MW2496 (*satf2581*) |  | NmrA-like family protein | 36.14 | 34.99 | 40.18 |
| MW2497 (*satf2582*) |  | Conserved hypothetical protein | 34.04 | 18.41 | 28.12 |
| MW2498 (*satf2583*) | *rob* | Transcriptional regulator | 47.86 | 29.38 | 20.46 |
| MW2499 (*satf2584*) |  | 2-deoxy-D-gluconate 3-dehydrogenase | 41.21 | 32.51 | 24.43 |
| MW2500(*satf2585*) |  | Amidohydrolase family protein | 22.13 | 34.51 | 18.32 |
| MW2501 (*satf2586*) |  | Putative hydrolase | 10.89 | 5.27 | 4.29 |
| MW2586 (*satf2686*) | *icaA* | intercellular adhesion protein A | 6.01 | 2.76 | 1.75 |
| MW2587 (*satf2687*) | *icaD* | intercellular adhesion protein D | 5.59 | 3.72 | 5.36 |
| MW2588 (*satf2688*) | *icaB* | intercellular adhesion protein B | 8.05 | 6.35 | 6.25 |
| MW2589 (*satf2689*) | *icaC* | intercellular adhesion protein C | 7.82 | 1.92 | 6.12 |

*^a^* Fold changes indicate increases in expression levels in TF2758 from ATCC49775. Experiments were repeated three times.
