# Supplementary figures and images for: A Novel Repressor of the ica Locus Discovered in Clinically Isolated Super-Biofilm-Elaborating Staphylococcus aureus
Source: mBio. 2017 Jan 31;8(1):e02282-16. doi: 10.1128/mBio.02282-16 (PMC5285506; doi:10.1128/mBio.02282-16)

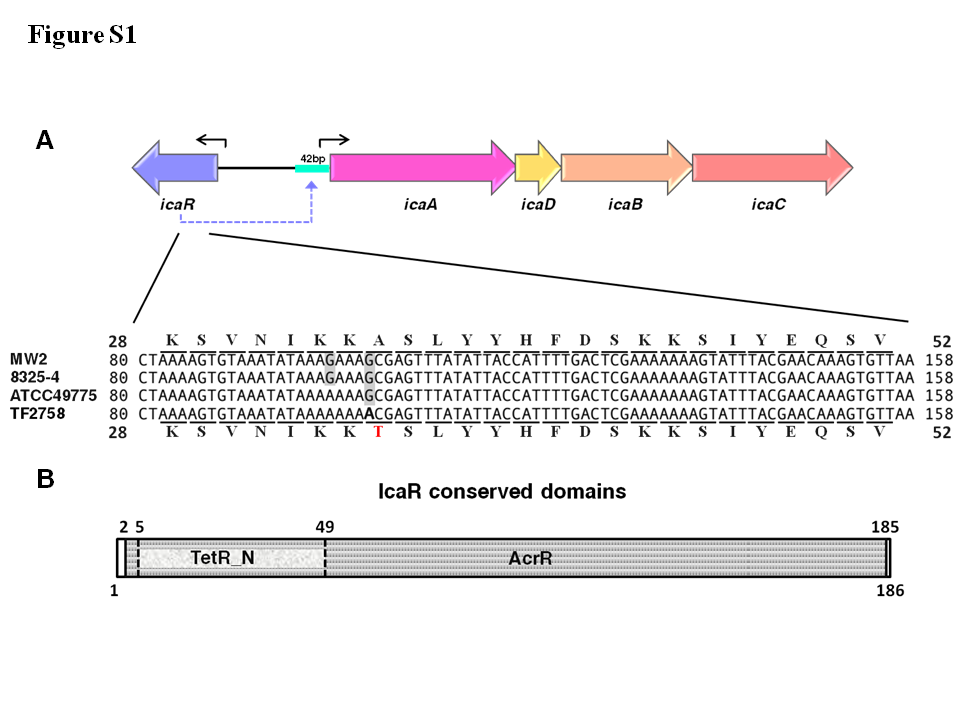

Supplement: FIG S1 [file mbo001173169sf1.tif]

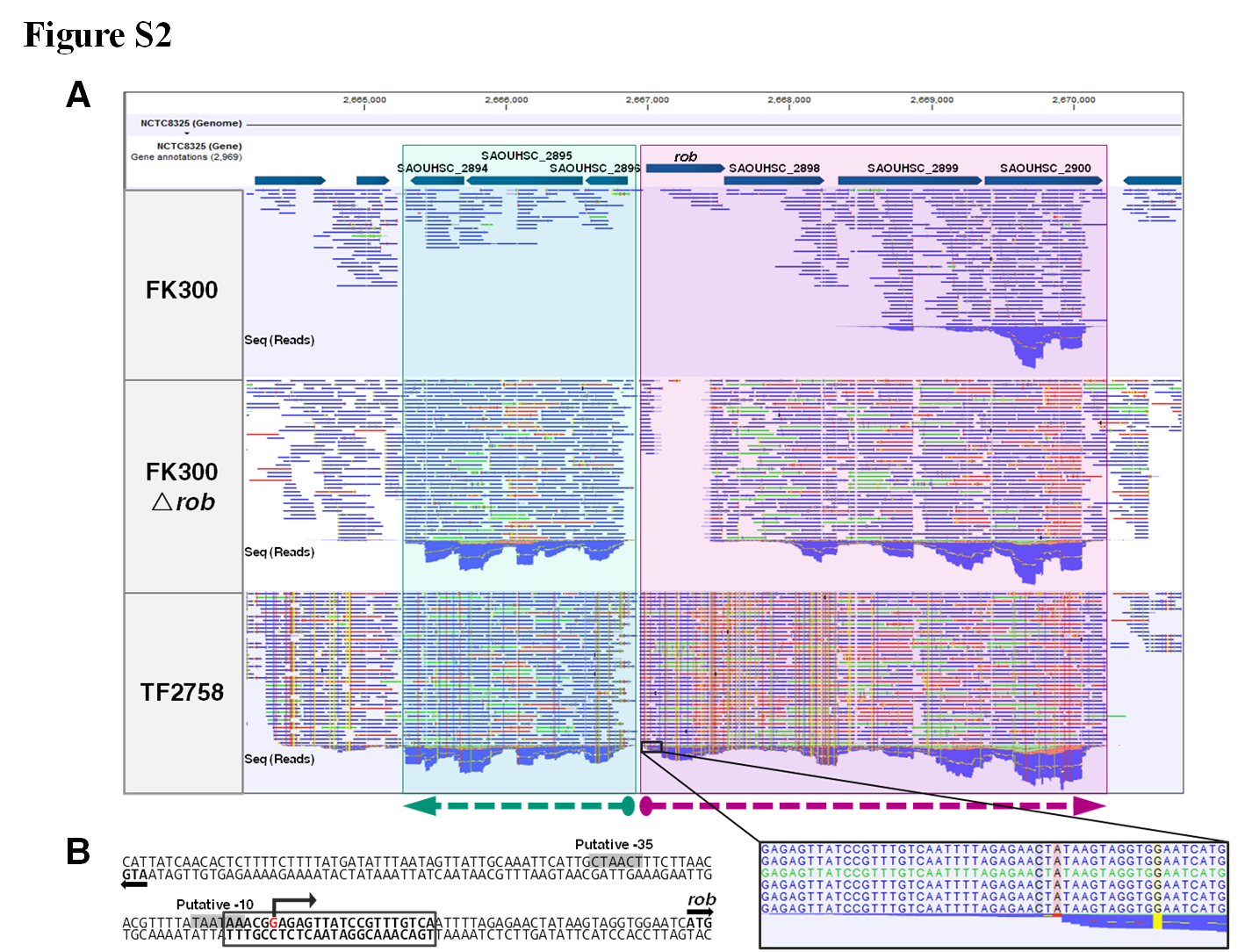

Supplement: FIG S2 [file mbo001173169sf2.tif]

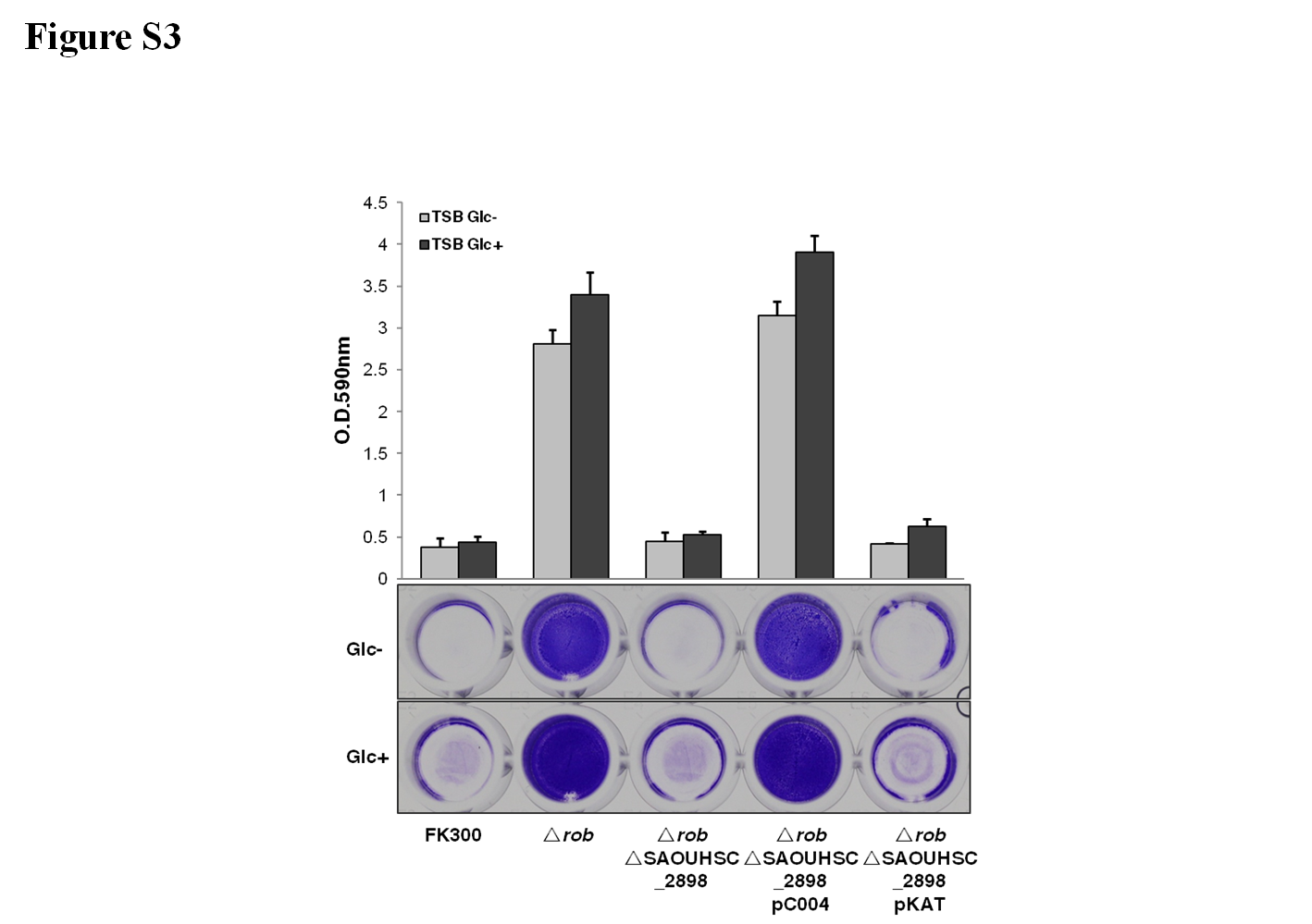

Supplement: FIG S3 [file mbo001173169sf3.tif]

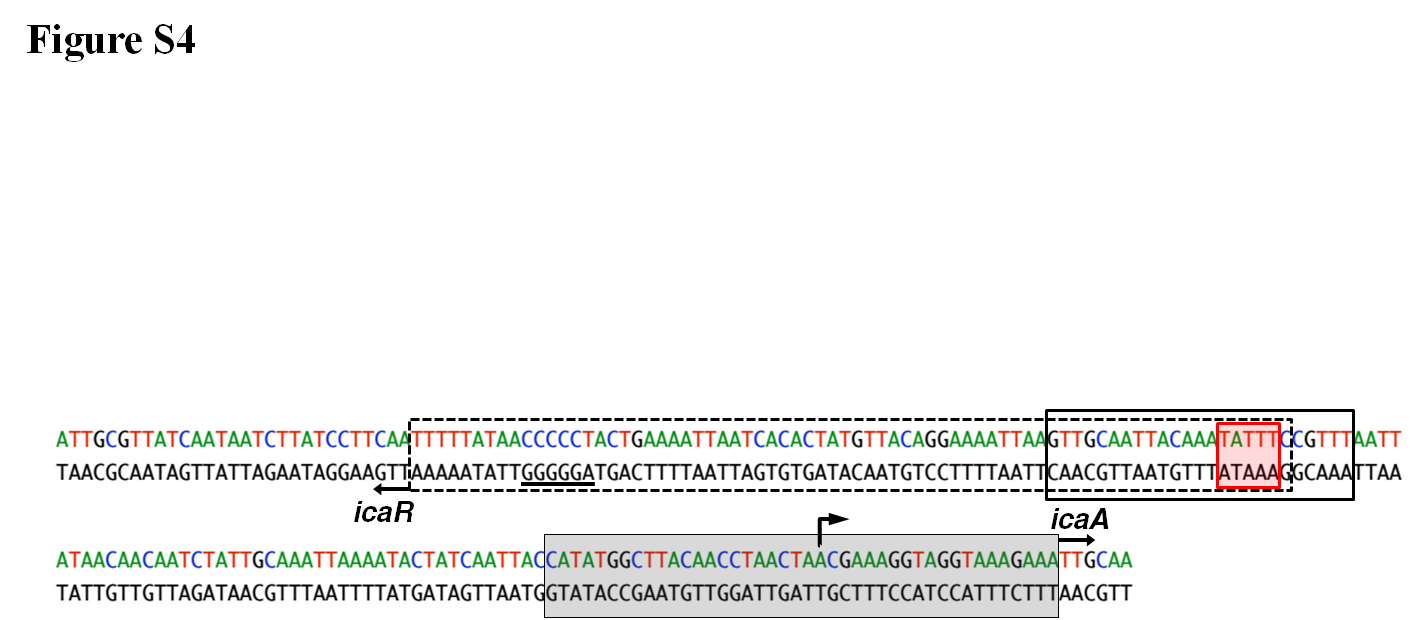

Supplement: FIG S4 [file mbo001173169sf4.tif]

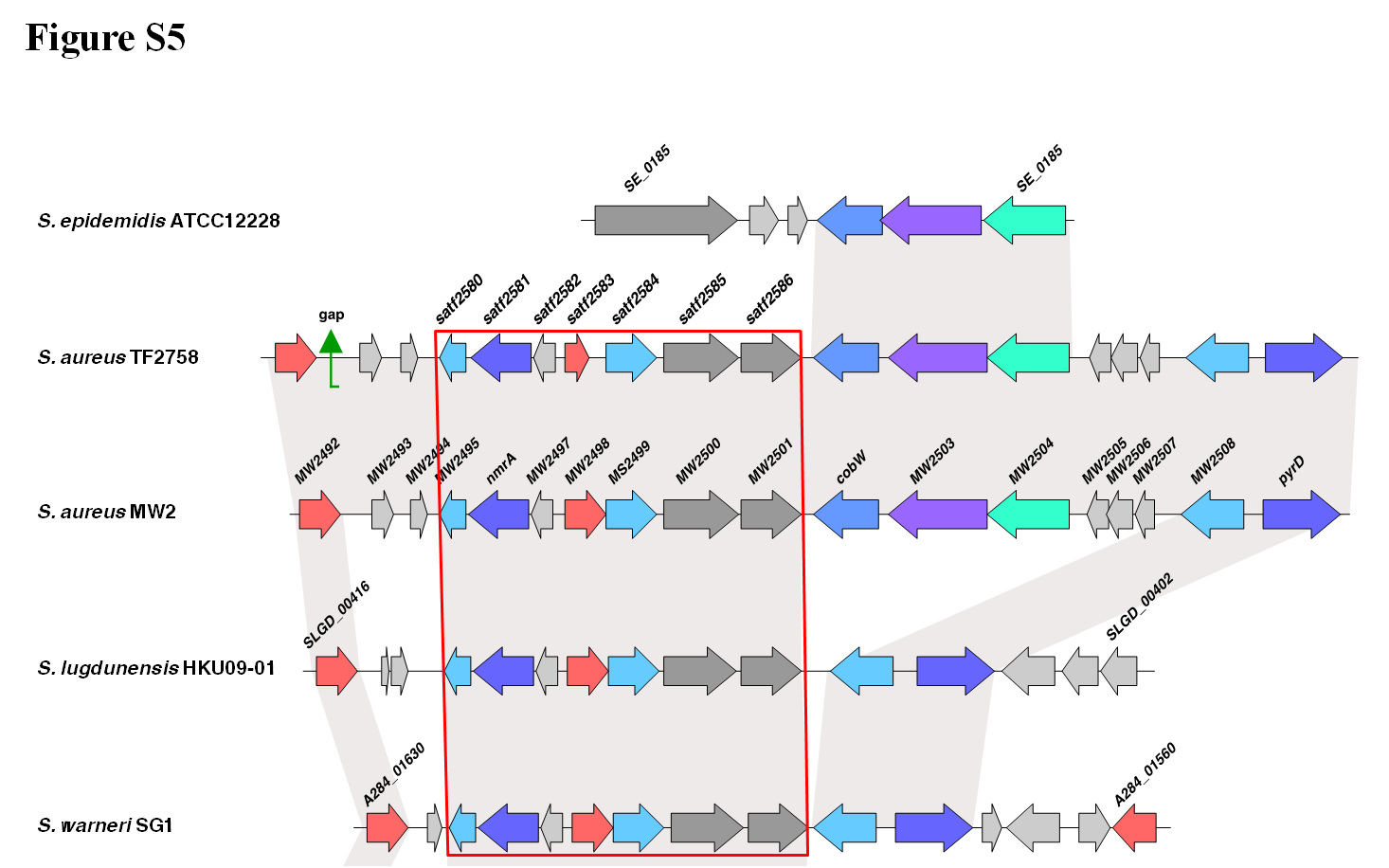

Supplement: FIG S5 [file mbo001173169sf5.tif]
